# Native plant diversity for ecological reclamation in Moroccan open-pit phosphate mines

**DOI:** 10.3897/BDJ.11.e104592

**Published:** 2023-07-11

**Authors:** Hamza Zine, Rachid Hakkou, Abdelhak Elmansour, Sara Elgadi, Ahmed Ouhammou, Mostafa Benzaazoua

**Affiliations:** 1 Geology and Sustainable Mining Institute (GSMI), Mohammed VI Polytechnic University, Ben Guerir, Morocco Geology and Sustainable Mining Institute (GSMI), Mohammed VI Polytechnic University Ben Guerir Morocco; 2 Innovative materials, energy, and sustainable development laboratory (IMED-Lab), Faculty of Sciences and Technology, Cadi Ayyad University, Marrakech, Morocco Innovative materials, energy, and sustainable development laboratory (IMED-Lab), Faculty of Sciences and Technology, Cadi Ayyad University Marrakech Morocco; 3 Laboratory of Microbial Biotechnologies, Agrosciences and, Environment (BioMAgE), Phytobiodiversity and Environment team, regional herbarium 'MARK', Faculty of Sciences Semlalia, Cadi Ayyad University, Marrakech, Morocco Laboratory of Microbial Biotechnologies, Agrosciences and, Environment (BioMAgE), Phytobiodiversity and Environment team, regional herbarium 'MARK', Faculty of Sciences Semlalia, Cadi Ayyad University Marrakech Morocco

**Keywords:** arid and semi-arid climate, biodiversity, ecology, local flora, marginal land, mining site, SDGs, sustainability

## Abstract

Mining activities have significant impacts on the environment, particularly in terms of the destruction of natural habitats andbiodiversity loss. With the increasing awareness of the importance of ecological restoration and conservation, there is a growing need to study and understand the flora that thrives in mining sites in order to facilitate successful reclamation efforts. This study aimed to investigate the floristic composition and plant diversity of four phosphate mine sites (PMSs) in Morocco, namely Bou Craa mine (BCM), Ben Guerir mine (BGM), Youssoufia mine (YSM), and Khouribga mine (KHM).

The study found a total of 215 vascular plant species from 166 genera and 49 taxonomic families across the four sites. BGM was the most diverse site with 120 plant species, followed by KHM with 75, YSM with 57, and BCM with 54. Compositae family species were the most common at BGM and KHM, while Amaranthaceae species were dominant at BCM, and Poaceae and Compositae at YSM. Therophytes (annual species) were the most common functional group (45.0%), followed by chamaephytes (19.6%) and hemicryptophytes (15.9%).

*Atriplexnummularia* and *Chenopodiumalbum* were the most common species found at all four sites, while *Atriplexsemibaccata*, *Bassiamuricata*, *Haloxylonscoparium*, and 12 other species were common at three sites. However, 156 plant species were found at only one site. The findings of this study highlight the significant abundance of plant species in Moroccan PMSs and provide a basis for successful ecological engineering rehabilitation plans. The study emphasizes the importance of studying the indigenous plant species that naturally populate these marginal lands to ensure successful reclamation efforts.

## Introduction

Since pre-historic times, open-pit mining for phosphate and other valuable geological materials has caused severe ecological disturbances worldwide (Kondratenko et al. 2022, Reta et al. 2018). In keeping with population growth and advancements in technology and science, the rate of consumption of mineral resources has continued to increase (Reta et al. 2018). Human societies require ever-greater mineral resources to satisfy this continuously increasing demand (Lima et al. 2016).

In most countries with longstanding mining industry operations, open-pit mining, which is the most common method of phosphate extraction, remains an acute issue from an environmental perspective (Batterhama 2014). This activity can cause a complete change in the natural landscape and the ecological system, including the destruction of biodiversity (Zine et al. 2020). Hence, this mining activity is known to perturb environmental homeostasis unless prevented by a well-planned rehabilitation strategy (Lottermoser 2011). To successfully rehabilitate the land after mining activities, it is crucial to revitalize the soil and re-establish the local vegetation. The presence of an initial plant cover is of clear importance in launching the ecological process of rehabilitation (Bochet et al. 2010). In light of increasing ecological awareness, spontaneous flora that were once viewed as ‘weeds’ have begun to gain attention for the variety of positive attributes they offer (Kondratenko et al. 2022). The native pioneers’ flora have the potential to control erosion in storage facilities, stabilize those facilities through a root net system and moderate floods, as well as enhance surface moisture and improve the content of organic matter (Bateman et al. 2021, Turisová et al. 2016).

Native flora is known to establish a self–sustaining vegetative cover on marginal land such as mine deposits and support the resilience and recovery of the historical ecosystem (Prach et al. 2013). According to Mukhopadhyay (2010), Sheoran et al. (2010) and Zine et al. (2021a), native plants are known for their fast growth, ability to easily establish themselves in stressed environments and high tolerance to harsh climatic conditions (Zhang et al. 2020). Using native plants to restore a landscape can help to reverse a species loss trend caused by harmful anthropogenic activities given that in the long run, these plant communities require little maintenance (Minas 2015). Additionally, they tend to resist damage from freezing, drought and common diseases (Antoniadis et al. 2017). Furthermore, there are specific relationships between mycorrhizae and plants, invertebrates and woody debris, pollinators and flowers and birds and structural habitats that only native plant species can rebuild (Carrasco et al. 2011).

Revegetation of mining sites in arid and semi-arid ecosystems should involve the use of plants that have been selected based on their ability to survive and regenerate or reproduce under severe conditions (Carrasco et al. 2011, Mendez and Maier 2008). Because of the above-mentioned characteristics of native plants, one common and effective restoration used strategy is the establishment of new populations of native plants (Abella et al., 2012). Batty (2005) indicates that in tandem with the previously mentioned benefits, the indigenous plants add scenic beauty, maintain the natural inheritance and furnish habitat for native wildlife.

The in-depth study of this particular type of flora is thus of special interest. To better understand the understudied native flora in PMSs, it is necessary to create an itinerary of the flora at these sites and highlight its diversity. Floristic surveys on mine sites provide relevant data about the sites’ floristic potential (Martínez-Ruiz et al. 2007, Pulchérie et al. 2018, Zine et al. 2021b). To restore the resiliency of these disturbed ecosystems, it is important to address as many aspects of native and spontaneous vegetation as possible.

The main objective of this study is to assess the diversity of spontaneous and native flora in phosphate mining sites located in the Saharan, arid, and semi-arid climates of Morocco, and to understand how these plants adapt to their challenging environment. Thus, the study of native phytodiversity at these mining sites is an important aspect of ensuring PMSs sustainability.

## Material and methods

### Site locations and study area

**Figure 1.**
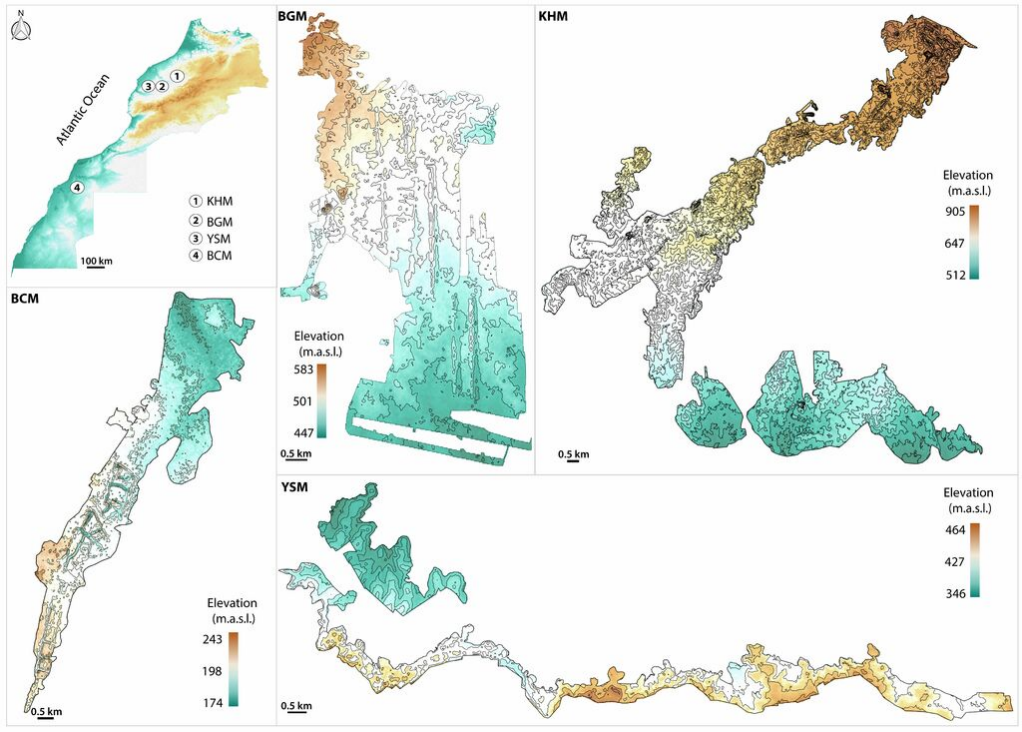
Location and elevation maps of the phosphate mines sites. BCM: Bou Craa phosphate mine; BGM: Ben Guerir phosphate mine; YSM: Youssofia phosphate mine; KHM: Khouribga phosphate mine.

This research investigates all four phosphate mining sites in Morocco: Bou Craa (BCM; 26°21’14.99”N; 12°48’37.69”W) in the south and Ben Guerir (BGM; 32°15’11.71”N; 07°49’04.40”W), Youssofia (YSM; 32°14’25.86”N; 08°23’55.04”W) and Khouribga (KHM; 32°45’50.25”N; 06°48’36.76”W) in the north (Fig. 1). Extensive surface mining of phosphate has been conducted in Morocco since 1912.

In fact, in 1920, the Office Chérifien des Phosphates company was the only company that mined Moroccan phosphates. The company became OCP Group in 1975. In 1921, phosphate extraction and treatment activity started in the Khouribga region. In 1931, underground extraction activity began in Youssofia, and the Ben Guerir mining site was launched in 1980. The phosphate mining activity in the south of Morocco – commonly referred to as the Bou Craa region – started in 1972, led by phosphate miner and refiner PhosBou Craa (OCP S.A. 2023).

### Climatic features

**Figure 2.**
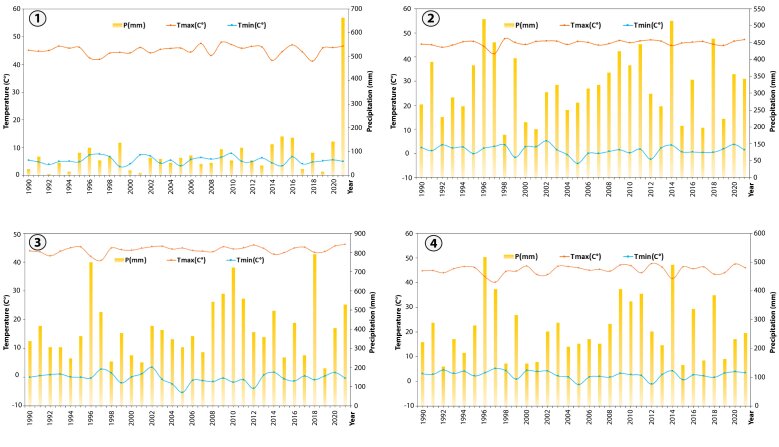
Climatic features of each mining site. (1) BCM: Bou Craa mine; (2) BGM: Ben Guerir mine; (3) KHM: Khouribga mine; (4) YSM: Youssofia mine.

**Figure 3.**
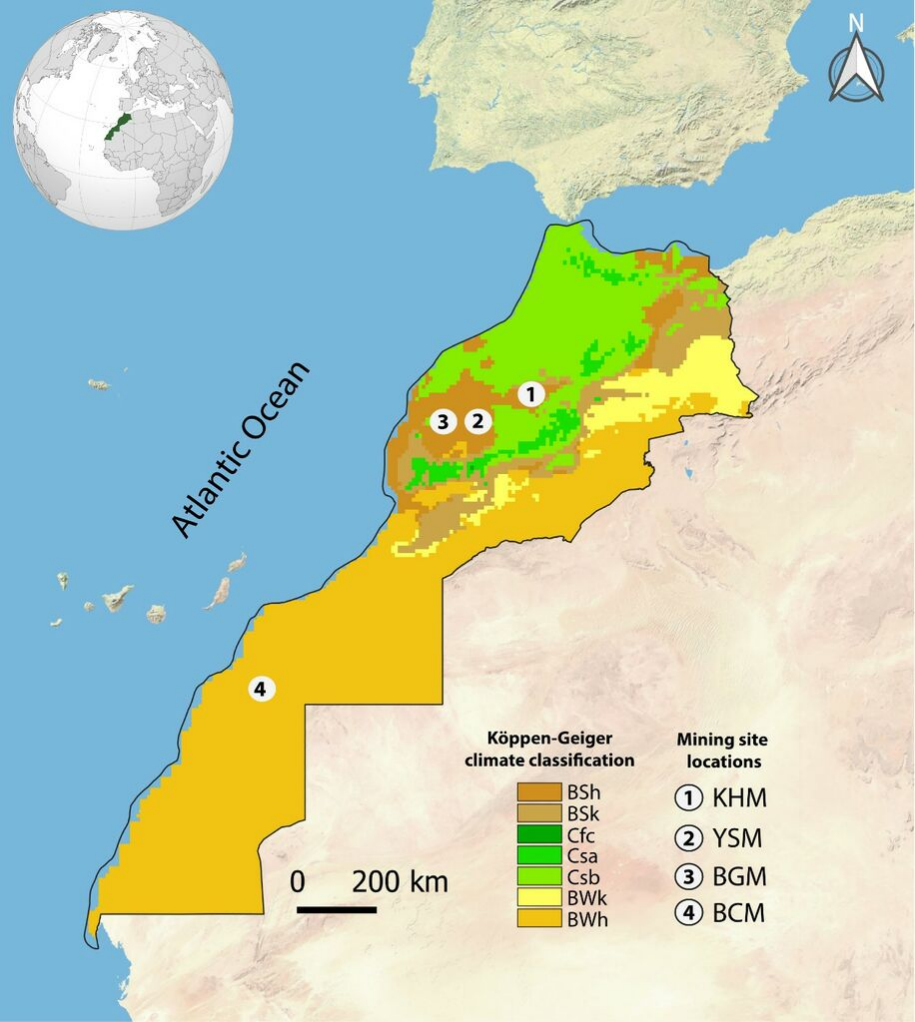
Position of the studied mining sites in Köppen-Geiger climate classification. BCM: Bou Craa phosphate mine; BGM: Ben Guerir phosphate mine; YSM: Youssofia phosphate mine; KHM: Khouribga phosphate mine. BSh: Arid steppe-hot arid; BSk: Arid steppe-cold arid; Cfc: Warm temperate-fully humid-cool summer; Csa: Warm temperate-summer dry- warm summer; Csb: Warm temperate-summer dry-warm summer; BWk: Arid desert-cold arid; BWh: Arid desert-hot arid.

Fig. 2 highlights the climatic features of each mining site. Climatic data for the period 1990–2021 were downloaded from https://power.larc.nasa.gov/. According to these data and to the Köppen climatic classification (Fig. 3), the BCM is in a region that features an arid climate (BWh), otherwise known as Saharan. This site in Morocco’s extreme south enjoys a unique Saharan climate characterized by a long dry period (May to December). The BGM and YSM are both characterized by a semi-arid climate (BSh). However, the KHM site is characterized by a semi-arid climate with a shift to the mild Mediterranean climate (Csa). Generally, at the BGM, YSM and KHM sites, seasonal rainfall is unevenly distributed with a dry season of 6 months.

### Plant inventory method

To have a scope and understand the vascular plant diversity and its taxonomic richness in the studied areas. Floristic and botanic surveys were carried out in 2021 and 2022 at the BCM, KHM, BGM and YSM. Due to seasonal factors, the field investigations began with the site located in the extreme south of Morocco (i.e., the BCM site), followed by KHM. Afterwards, the BGM and YSM sites were investigated as well. At each location, the creation of a botanical checklist was performed in a way that optimized the observations and ensured a maximum of records on the whole flora of the studied site.

The field surveys involved direct observation and the collection of herbarium plant specimens of unknown plants. These herbarium specimens offered valuable information about the distribution and taxonomy of plant species over time in these mining areas. The nomenclature that this article adopts is that of the ‘Flore Pratique du Maroc’ (Fennane et al. 1999, Fennane et al. 2007, Fennane et al. 2014), which was updated in accordance with the Angiosperm Phylogeny Group (APG VI et al. 2016).

The inventoried plant species’ functional groups were also studied. The Raunkiaer’s life-form system features the simplest and most effective proxies that represent the botanical and ecological adaptations and habitat requirements of plants. The collected plant species were classified into six main life-form categories: Phanerophyte, Nano-phanerophyte, Chamaephytes, Hemicryptophyte, Geophyte and Therophyte. The determination of the life-form for each plant species enabled the calculation of the proportion of various life-forms within the flora at the studied sites, which is referred to as the biological spectrum. This spectrum is useful primarily because it reflects the climatic conditions of the surrounding environment through the structure of the vegetation of which it mirrors.

## Statistical analysis and software

Statistical differences between the diversity of each phosphate mining site were assessed as follows. First, a one-way analysis of variance (ANOVA) was used. Second, Tukey’s post-hoc test was used to determine the significant difference. Correlations and clustering and Factorial compounds analysis (FCA) were performed to highlight the relation between flora and mine sites using Python 3.11 (December 2022) and R 4.0.3 software (R Core Team, 2020) and Corrplot and Circlize packages. The maps were generated using Q GIS 3.16.9-Hannover (2020).

## Results and discussions

### Floristic analysis

Despite the severe climatic conditions at the studied mining sites, which are characterized by their semi-arid-to-Saharan bioclimate, the results showed intense floristic richness at the sites. The inventory taken at each site allowed for the creation of a complete list of the flora present in the mining areas. In total, 215 vascular plant species were inventoried. Table 1 provides a global list of plant species inventoried at the PMSs.

**Figure 4.**
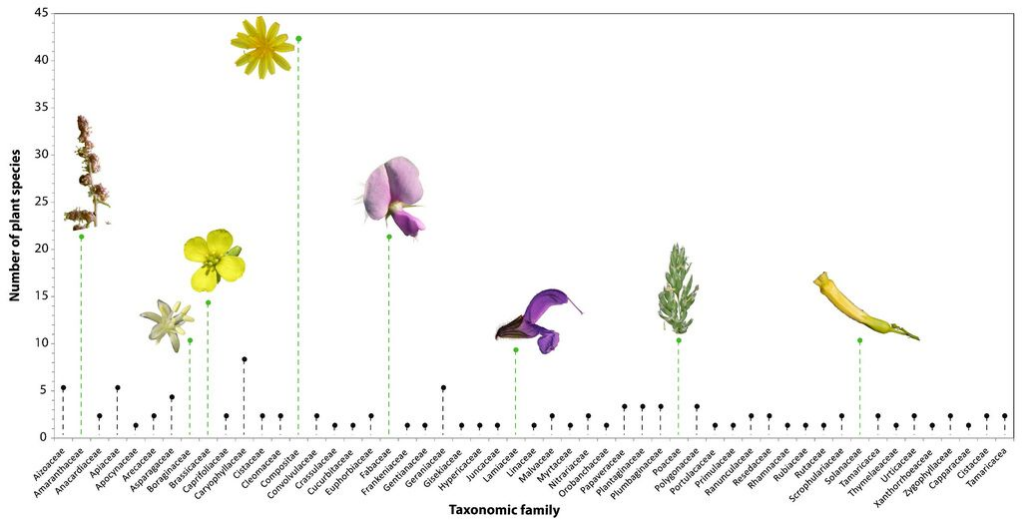
Global taxonomic family diversity and their specific richness in the phosphate mining sites in Morocco.

Globally, a total of 49 taxonomic families were identified, which is quite a large number. The main plant families that colonised the PMSs are as follows (Fig. 4). First, the ubiquitous Compositae family was the most common, with more than 42 plant species. In second and third place were the Fabaceae and Amaranthaceae families, both of which were represented by more than 21 vascular plant species at the sites. Finally, the fourth and fifth most common plant families were Brassicaceae and Poaceae, respectively.

**Figure 5.**
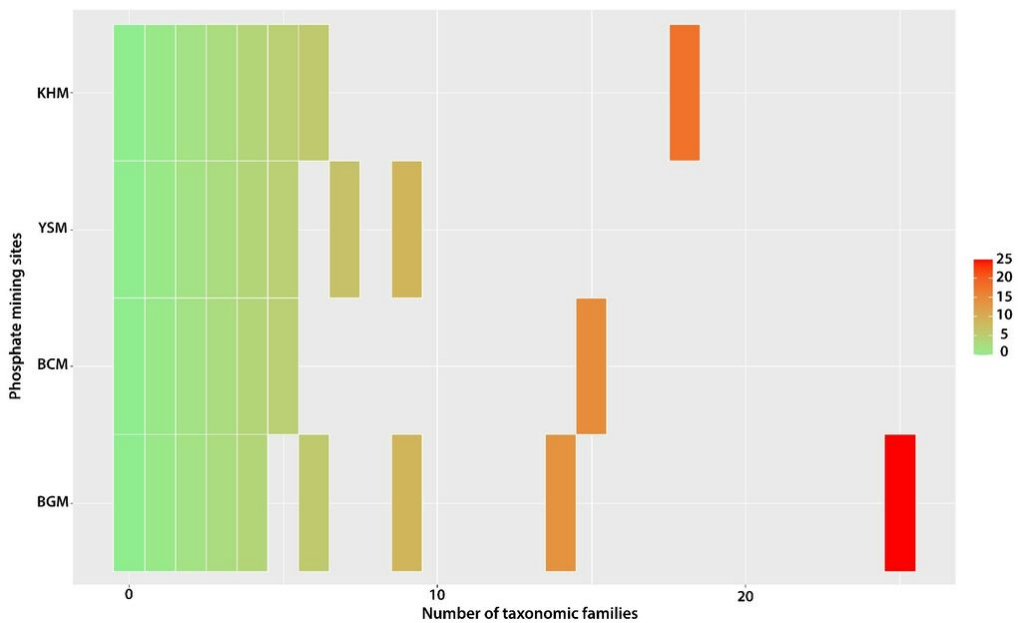
Taxonomic family’s richness per site. BCM: Bou Craa phosphate mine; BGM: Ben Guerir phosphate mine; YSM: Youssofia phosphate mine; KHM: Khouribga phosphate mine.

**Figure 6.**
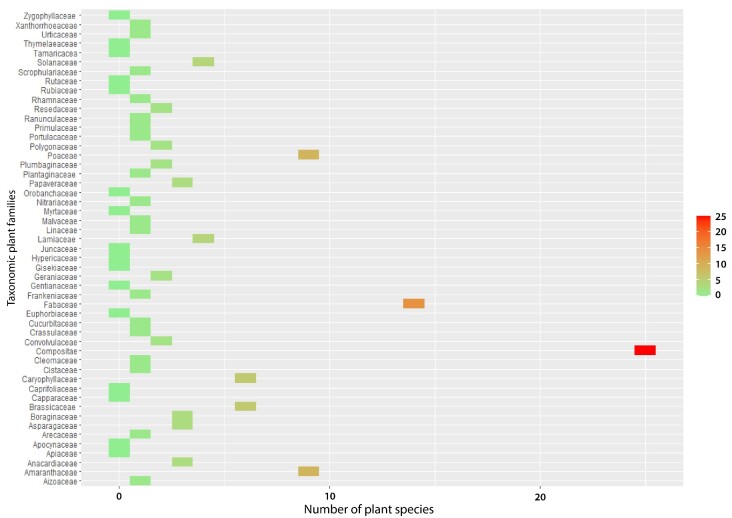
Number of plant species by taxonomic family at Ben Guerir phosphate mine.

**Figure 7.**
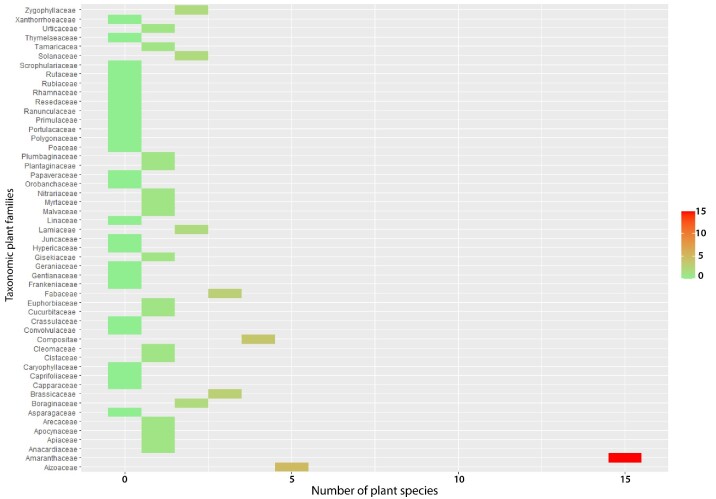
Number of plant species by taxonomic family at Bou Craa phosphate mine.

**Figure 8.**
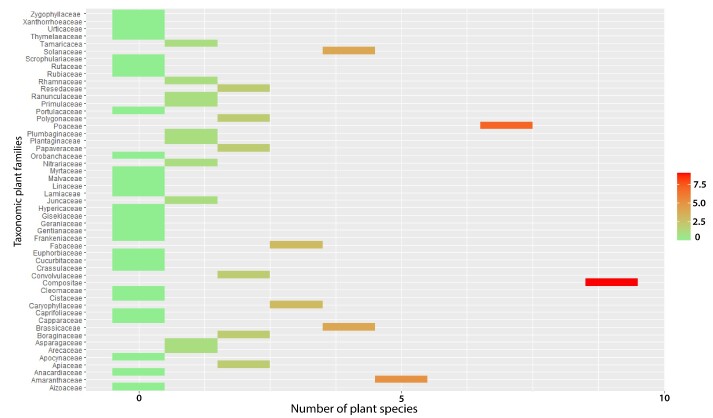
Number of plant species by taxonomic family at Youssofia phosphate mine.

**Figure 9.**
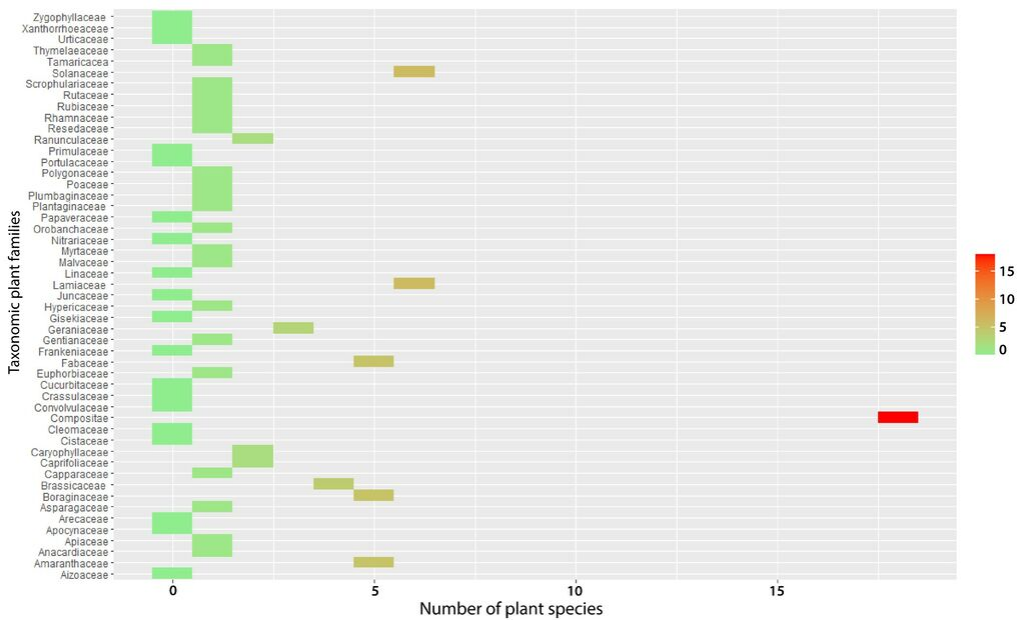
Number of plant species by taxonomic family at Khouribga phosphate mine.

At the BGM, 120 vascular plant species from 103 genera and 35 families were recorded. Compositae was the dominant family with 25 plant species (20.8% of the total species), followed by Fabaceae (14; Fig. 5). The BGM was the most diverse site among the four mines, with 25 taxonomic families (Fig. 5). This area is characterized by a steppic formation of *Zizyphuslotus* Lam. as a climatic plant species vegetation. At the KHM, 75 vascular plant species from 34 genera and 32 families were inventoried. Compositae was the dominant family with 18 plant species (22.78% of the total species), followed by Lamiaceae with 6 plant species (7.6%). This site occupied the second rank in terms of taxonomic family diversity (Fig. 5). The KHM area is characterized by a shrub plant formation of *Chamaeropshumilis* L. as a climatic plant species vegetation. The third most diverse site was YSM (Fig. 8), with 57 vascular plant species from 52 genera and 24 families. Compositae and Poaceae shared the first rank, with 7 plant species for each, comprising 12.08% of the total plant species. In the second position was Amaranthaceae, with 8.8% of the inventoried flora. The YSM area is characterized by a shrub-steppe of *Chamaeropshumilis* L.. The BCM site, which is a wooded steppe of Acacia tortilis subsp. raddiana *(Savi) Brenan*, was slightly less diverse than the YSM site. A total of 54 vascular plant species belonging to 46 genera and 25 taxonomic families were inventoried at the site (Figs 6, 7, 8, 9).

### Functional groups and biological spectrum

Regarding the life-forms of the identified species, Fig. 11 outlines the biological spectrum at the studied sites. From most to least common, those life-forms are as follows: therophytes (annual plants; 45.0% of the total species), chamaephytes (shrubs and bushes; 19.6%), phanerophytes and hemicryptophytes (19.2%) and geophytes (3.3%). This reflects their dominance at each mine site. At all the studied sites, therophytes were the most dominant life-form; they are represented by 53.6%, 57.5%, 53.6% and 43.0% at BCM, BGM, YSM and KHM, respectively. Additionally, chamaephyte were also observed to be blooming at the studied mining sites, with 37.0% at BCM, 14.2% at BGM, 10.7% at YSM and 16.5% at KHM. The phanerophytes were poorly represented by nanophanerophytes.

### Plant composition analysis

**Figure 10.**
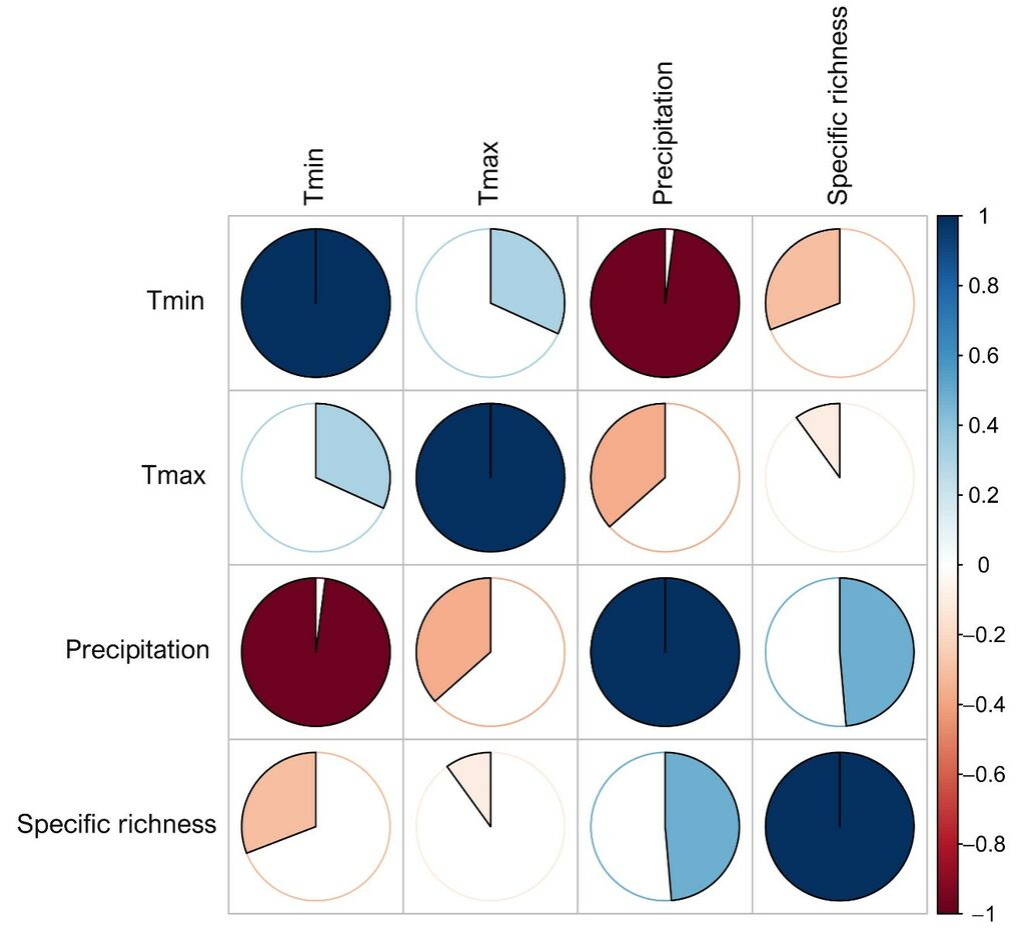
Pearson’s correlation of the maximal temperature (Tmax), minimal temperature (Tmin), precipitation and the global specific richness.

**Figure 11.**
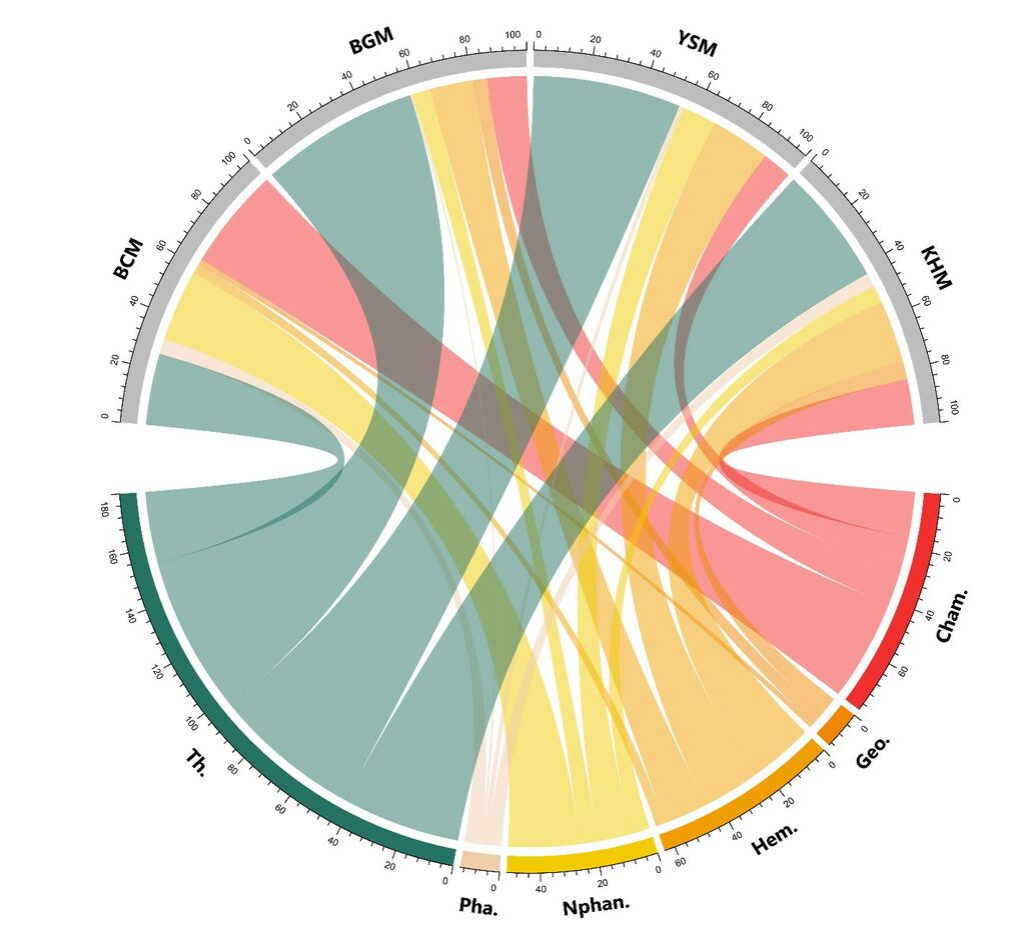
Biological spectrum of the flora of the phosphate mining sites in Morocco. Pha.: Phanerophyte; Nphan.: Nanophanerophyte; Hem.: Hemicryptophyte; Cham.: Chamaephyte; Geo.: Geophyte; Th.: Therophyte. BCM: Bou Craa phosphate mine; BGM: Ben Guerir phosphate mine; YSM: Youssofia phosphate mine; KHM: Khouribga phosphate mine.

**Figure 12.**
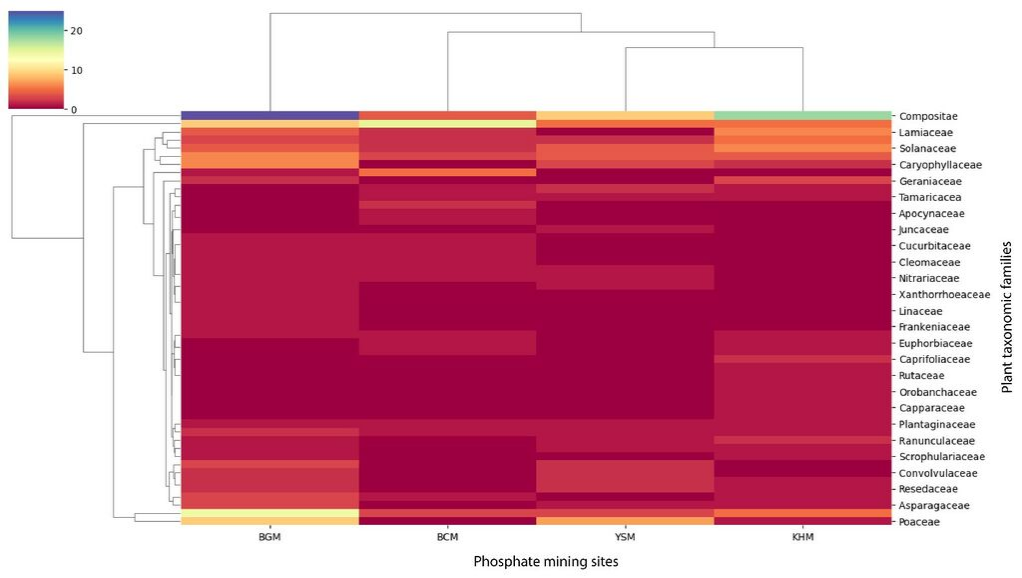
Heatmap clustering of the taxonomic plant family richness and the explored phosphate mining sites in Morocco. BCM: Bou Craa phosphate mine; BGM: Ben Guerir phosphate mine; YSM: Youssofia phosphate mine; KHM: Khouribga phosphate mine.

The composition of spontaneous species differed between the sites (Fig. 12). Each site’s climatic and geographical features, such as latitude, longitude, altitude and consequently the type of climate, influence not only species richness and community diversity but also how species cohabit and distribute themselves Fig. 10. On the one hand, there were some generalists that dominated almost all the PMSs, including *Chenopodiumalbum* L., *Atriplexsemibaccata* R. Br, *Bassiamuricata* (L.) Asch., *Haloxylonscoparium* Pomel, *Calendulaarvensis* M.Bieb., *Diplotaxistenuisiliqua* Delile, *Herniariahirsuta* L., *Launaeaarborescens* Murb., *Scolymushispanicus* L., *Anacyclusvalentinus* L., *Limoniumlobatum* Kuntze, *Rumexvesicarius* L., *Resedalutea* L., *Ziziphuslotus* Lam., *Nicotianaglauca* R.C. Graham and *Withaniafrutescens* Pauquy, Foleyolabillotii Maire.

On the other hand, some species showed a distinct inclination toward specific mine sites. At the BCM site, *Calotropisprocera* W.T. Aiton, *Forsskaoleatenacissima* L., *Fagoniazilloides* Humbert, *Tetraenagaetula* Beier & Thulin, *Hyoscyamusmuticus* L., *Saharanthusifniensis* Crespo & Lledó, *Cotulacoronopifolia* L., *Brocchiacinerea* Vis., *Zillaspinosa* Prantl, *Crotalariasaharae* Coss. and *Frankeniapulverulenta* L were observed. At BGM, *Marrubiumalysson* and *Haloxylonscoparium* Pomel were recorded. *Juncusacutus* L. and the abundant *Tamarixaphylla* H.Karst appeared at YSM. Lastly, at KHM, *Daphnegnidium* L., *Drimiaundata* Stearn, *Capparisspinosa* L. and *Chamaeropshumilis* L. were inventoried.

We can attribute the dominance of Compositae at all the mine sites except BCM, where Amaranthaceae was most common, to their adaptation strategies. These strategies have allowed Compositae to flourish in a large array of climatic conditions, especially those of the studied mining areas. Indeed, plant species belonging to the Compositae family have specific strategies to increase their reproductive success (Gutterman 1994). The achenes (fruits) of these plants feature a tuft of hairs called *pappus*; this structure increases the dispersal distances of the plants’ seeds (Sádlo et al. 2018). In addition, most plants in this family produce seed or fruit shapes with different germination behaviours, which seems to be an effective adaptive strategy in unpredictable arid, semi-arid and Saharan environments (Li et al. 2022). Another strategy that these plants use to reduce the effects of environmental conditions and colonize large areas is to retain mature seeds on a dead mother plant for an extended period; these dead plants act as long-term protected seed banks. Also, the wide ecological distribution of the plant species belonging to Poaceae can be attributed to their ability to adapt to challenging environments and their effective dispersal strategies through wind of their diaspores. Moreover, the thriving presence of this family can be traced to their anatomical adaptations, particularly their thick epidermal cell walls, bolstered by abundant sclerenchyma tissue (Banan et al. 2019).

After dispersal, Compositae seeds are harvested and eaten by insects, birds and other animals. Species that protect their seeds thus have a survival advantage. In some species inventoried at the studied sites, such as those belonging to the *Atriplex* and *Bassia* genera, dead or old mother plants termed ‘nurse plants’ act as the species’ most important seed banks; they periodically release some of their seeds during rainfall events over a period of several years (Filazzola and Lortie 2014, Padilla and Pugnaire 2006). Annual species that inhabit stressed and marginal areas frequently disperse seeds by umbrohydrochory and anemochory, the latter of which is a special form of seed dispersal that occurs mainly in environments where rainfall plays a determinative role in plant blossoming and life cycles (Thompson et al. 2008). In addition, in the harsh environment of a phosphate mining site, therophytes (annuals) adopt a fractional germination approach that allows them to persist in these difficult conditions where survival is highly variable from year to year (Filazzola and Lortie 2014). The overall floristic list of the four mines reveals an abundance of therophytes, also known as annual herbaceous species, which reflects the sites’ local arid, semi-arid and Saharan bioclimates (Zine et al. 2021b).

In addition to the Compositae family, Poaceae and Fabaceae (Leguminosae) are known to constitute the lion's share of plant species in arid and semi-arid areas. The Compositae family, also known as the Asteraceae family (notably, the largest family on the list), is not only the largest family in the Flore de Maroc (Fennane and Ibn Tattou 2012), but also the largest and most widespread family of flowering plants in the world (WFO 2023). The family’s prevalence can be attributed to these plants’ tolerance to a wide range of ecological conditions and efficient seed dispersal capability. An eminent feature of the floristic composition of the flora at the four mining sites is the floristic importance of a few families; most plant species belong to a limited number of plant families such as, Anacardiaceae, Apiaceae, Apocynaceae, Arecaceae, Cistaceae, Cleomaceae, Cucurbitaceae, Malvaceae, Myrtaceae, Plantaginaceae, Plumbaginaceae, Tamaricaceae, Euphorbiaceae, Gisekiaceae, Nitrariaceae, Urticaceae and others. In the present study, 52%, 64%, 42% and 67% of the total number of plant families inventoried at BGM, BCM, YSM and KHM, respectively, were represented by only a single species. This is a common characteristic of flora in harsh environments. It is thought that this indicates that only a small number of the many species in these ancient plant families have adapted and survived in harsh environments, while the other species that failed to survive have become scarce.

### Local plant diversity: a promising avenue for sustainable mining reclamation

To individualize the species specific to each inventoried mining site, and to highlight their potential in the rehabilitation of PMSs in Morocco, we used Factorial Component Analysis (FCA) in connection with the qualitative data used, namely, the floristic list, their uses cited in the literature, as well as the PMSs inventoried. The FCA allows the identification of links, dependencies, and matches between the variables of the data matrix. For this purpose, we have prepared a table S1 that involves all the plant species, their cited use, and the four PMSs Suppl. material 1.

**Figure 13.**
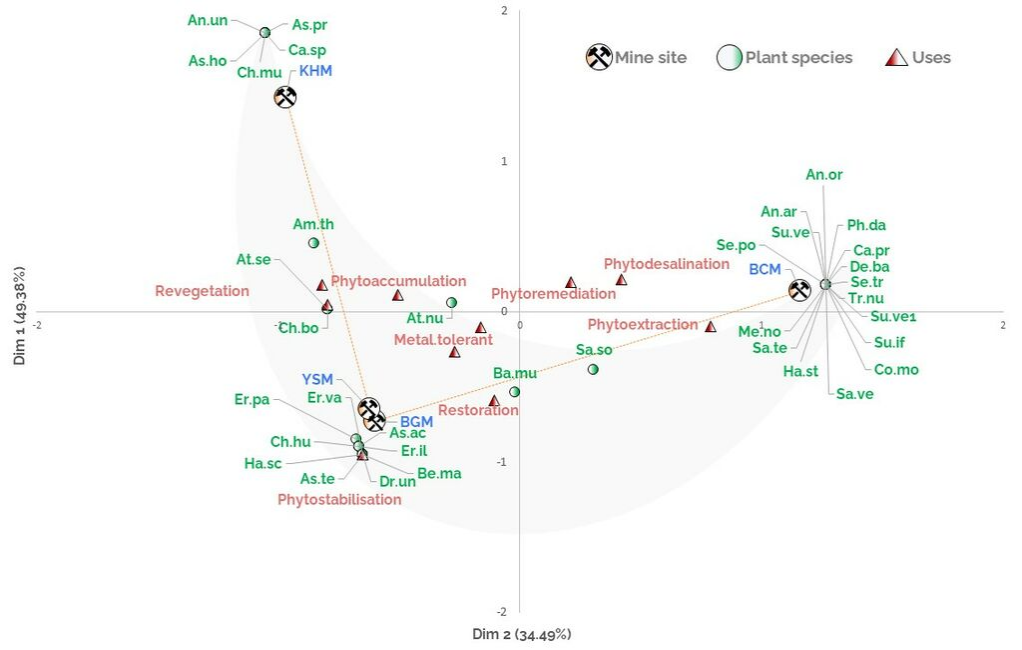
Factorial compounds analysis of the plant species and their uses in the inventoried mining sites. BCM: Bou Craa mine site; BGM: Ben Guerir mine site; YSM: Youssofia mine site; KHM : Khouribga mine site. The data used for the FCA analysis is provided in supplementary material.

The eigenvalues, corresponding to the inertia of the scatter plot along each axis, are relatively high. They provide information on a block partition structure. The factorial plane formed by the first two dimensions represents 83.87% of the total inertia of the table analyzed, with a predominance of dimension 1 (49.38%) and the expression of the Guttman effect (Fig. 13).

Analysis of the dimension 1 placed the KHM, YSM, and BGM on the negative side. However, the BCM is placed on the positive one. The distribution of PMSs was associated to the modalities of plant uses, such as revegetation, phytostabilization, phytoaccumulation, metal tolerance and restoration on the negative side. Yet, phytodesalination and phytoextraction were linked to BCM on the positive side.

Analysis of the dimension 2, records plant species for each mine site. In the positive side, we distinguish two different plant communities specific to the KHM on the negative side of Dim1 and BCM on the positive side of Dim1. The KHM plant community is composed essentially by *Anchusaundulata* L.; *Capparisspinosa* L. and *Chenopodiummurale* L. For the BCM, the Dim 2 highlights the community composed of *Anabasisoropediorum* Maire; *Calotropisprocera* (Aiton) W.T. Aiton; *Deverrabattandieri* (Maire) Podlech; *Searsiatripartita* (Ucria) Moffett. According to the same axe, the YSM and BGM share similar plant community composed, mainly, by *Bassiamuricata* (L.) Asch., *Haloxylonscoparia* (Pomel) Il'in; *Eryngiumilicifolium* Lam.; *Drimiaundata* Stearn. On the other hand, Dim2 also emphasizes the plant species that colonised more than two mine sites, which are promising candidates for the revegetation of the PMSs, such us *Amaranthusthunbergii* Moq.; *Atriplexsemibaccata* R. Br., *Chenopodiumboscianum* Moq., that more specialized in revegetation and phytoaccumulation, and *Bassiamuricata* (L.) Asch. and *Salsolasoda* L. that have been cited in restoration and phytoextraction research studies (Fig. 13).

Plant succession following the external disturbance that open-pit mining causes has ecological and practical interests. During plant rooting at different stages of succession, the recolonization of varying plant species plays an essential role in the soil-formation process, promoting vegetation succession by improving soil conditions (Burga et al. 2010). The success of ecosystem restoration is assessed in terms of the rate of natural or spontaneous vegetation regeneration and soil nutrient composition.

In semi-arid and arid Mediterranean conditions, soil stabilization on top of mining waste is often achieved through the use of commercial non-native seed mixtures (Emam 2016, Jones 2003). However, the ability of these species to provide rapid vegetation cover over an exposed substrate in an environment with scarce seasonal rainfall is still questioned, especially in harsh environments like the ones at the PMSs in Morocco (Salinas and Casas 2007).

Nitrogen-fixing legumes are recognised as crucial components of natural succession. These species are critical since the associated rhizobial symbioses serve as a source of nitrogen in an ecosystem (Bechtaoui et al. 2019).

Additionally, according to Corlett (2020) local plant diversity increases the diversity of the ecosystem’s ability to absorb and store carbon. Areas with a diverse array of plants offer greater potential for carbon storage because different plant species have different growth patterns, root systems and nutrient requirements. Furthermore, an area with a diverse range of local plants will be more resilient to environmental stressors such as droughts and pest outbreaks, which can affect carbon sequestration (Ghorbanalizadeh and Akhani 2022). In addition to carbon sequestration, local plant diversity also has a crucial role in climatic awareness (Kaye and Quemada 2017). The presence of a plant diverse range in an ecosystem increases its resilience to changing climatic conditions, as different species are better adapted to changing conditions (Ren et al. 2016). This resilience is important to maintain an ecosystem’s stability and the services provided, such as water regulation, pollination, and soil stabilization. However, the introduction of invasive species in the rehabilitation of mining sites (Sheley et al. 2005) often complicates restoration and rehabilitation projects due to their toxicity and invasive characteristics (Jelena et al. 2016). Invasive species can make more difficult the restoration of local flora, such as the *Nicotianaglauca* Graham, which was observed at all the PMSs in Morocco. As such, it is important to raise awareness about the appropriate management of invasive plant communities. It is also crucial to prioritize the restoration of local plant diversity to ensure ecosystems’ continued functioning and continued ability to provide crucial ecosystem services (Andrews and Broome 2006). The use of indigenous plant species (i.e., those naturally found in the local area) can support the success of restoration efforts and improve long-term sustainability. Local plants are well adapted to the local soil and climatic conditions, making them more likely to thrive at the mining site. This, in turn, provides a more stable and sustainable ecosystem for other species to establish themselves and flourish. Moreover, incorporating local plants can help to maintain local genetic diversity, which is essential for the survival of many species (Kettenring et al. 2014). Local plants can also play an important role in reducing the risk of soil erosion as well as in stabilizing slopes, which can prevent runoff and minimize the spread of pollutants. Likewise, these plants establish themselves quickly and do not require irrigation, which decreases maintenance costs and could guarantee the sustainability of the reclamation project (Suleiman et al. 2011). Hence, in future planting design efforts, learning how to manage spontaneous vegetation to enhance both ecological and social values may be a more sustainable strategy than attempting to restore only historical ecosystems (Tredici 2010), especially in the face of climate change. Furthermore, besides their ecological functions, they have high aesthetic value as well (Li et al. 2019).

## Conclusions

Mining sites are known for the significant impact they have on the natural environment, including the fragmentation of plant habitats and biodiversity loss. However, it is interesting to note that despite the harsh and disturbed conditions that characterize these sites, many species of vascular plants can adapt in these areas. The phosphate mining sites in Morocco host an extraordinarily diverse range of plants, with 215 vascular plant species from 166 genera and 49 taxonomic vascular plant families. This diversity of plant life at Moroccan mining sites offers researchers a unique opportunity to study the resilience of plant species and their ability to colonize disturbed areas.

In summary, the results argued that *A.semibaccata*, Bassiamuricata and Salsolasoda are more resilient to colonize the majority of PMSs. However, some species have preference and affinity for colonizing particular sites more than others. For instance, the BCM is preferred, mainly, by *C.procera*, *S.tripartita* and *D.battandieri*, BGM and YSM are both colonized mainly by *H.scoparia*, *E.ilicifolium* and *D.undata*, the *C.spinosa*, *A.undulata* and *C.murale* showed preference to KHM. Therefore, these plant species are advised as the best candidates for the revegetation of the PMSs. These plants are often able to survive in conditions with limited soil depth, high levels of heavy metals and moisture and temperature fluctuations.

Further research is urgently needed to better understand the ecology of these unique ecosystems and the factors that drive plant diversity at mining sites. Additionally, comparative studies of spontaneous plant succession in mining areas can provide crucial information about vegetation dynamics that could help ensure the success of future reclamation programs, beginning with the use of locally collected seeds in future field experiments.

## Supplementary Material

AE3C5DE7-1755-5997-B97F-F3D986DB0C4B 10.3897/BDJ.11.e104592.suppl1 8142220 Supplementary material 1 Table S1 Data type Data used for the Factorial compounds analysis of the plant species and their uses in the inventoried mining sites File: oo_832652.xlsx https://binary.pensoft.net/file/832652 Zine Hamza, Hakkou Rachid, El Mansour Abdelhak, Elgadi Sara, Ouhammou Ahmed, Benzaazoua Mostafa

## Figures and Tables

**Table 1.** Global floristic list of plant species inventoried in phosphate mines in Morocco. Ph.: Phanerophyte; Nph.: Nanophanerophyte; Hem.: Hemicryptophyte; Ch.: Chamaephyte; G.: Geophyte; Th.: Therophyte. ANN: Annual; PER: Perennial.

**Taxonomic family**	**Latin name**	**Life form**	**Life span**
**Aizoaceae**	*Aizoanthemopsishispanica* (L.) Klak	Th	ANN
	*Mesembryanthemumcrystallinum* L.	Th	ANN
	*Mesembryanthemumnodiflorum* L.	Th	ANN
	*Sesuviumportulacastrum* (L.) L.	Hem	PER
	*Amaranthusthunbergii* Moq.	Ch	PER
	*Anabasisaretioides* Coss. & Moq. ex Bunge	Ch	PER
	*Anabasisoropediorum* Maire	Ch	PER
	*Atriplexnummularia* Lindl.	Ch	PER
	*Atriplexsemibaccata* R. Br.	Ch	PER
	*Bassiamuricata* (L.) Asch.	Th	ANN
	*Beta macrocarpa Guss*.	Th	ANN
	*Chenopodiumboscianum* Moq.	Th	ANN
	*Chenopodiummurale* L.	Hem	PER
	*Cornulacamonacantha* Delile	Nph	PER
	*Halocnemumstrobilaceum* (Pall.) M. Bieb.	Nph	PER
	*Haloxylonscoparia* (Pomel) Il'in	Ch	PER
	*Salsolasoda* L.	Th	ANN
	Salsola tetragona Delile	Ch	PER
	*Salsolavermiculata* L.	Ch	PER
	*Suaedaifniensis* Caball. ex Maire	Ch	PER
	*Suaedavera* Forssk. ex J. F. Gmel.	Nph	PER
	*Suaedavermiculata* Forssk. ex J. F. Gmel.	Ch	PER
	*Traganumnudatum* Delile	Ch	PER
**Anacardiaceae**	*Searsiatripartita* (Ucria) Moffett	Nph	PER
**Apiaceae**	*Carum* sp.	Hem	PER
	*Deverrabattandieri* (Maire) Podlech	Hem	PER
	*Eryngiumilicifolium* Lam.	Th	ANN
	*Eryngiumpalmatum* Pančić & Vis.	Hem	PER
	*Eryngiumvariifolium* Coss.	Hem	PER
**Apocynaceae**	*Calotropisprocera* (Aiton) W.T. Aiton	Nph	PER
**Arecaceae**	*Chamaeropshumilis* L.	Nph	PER
	*Phoenixdactylifera* L.	Ph	PER
**Asparagaceae**	*Asparagusacutifolius* L.	Nph	PER
	*Asparagushorridus* L.	Ch	PER
	*Drimiaundata* Stearn	G	PER
**Asphodelaceae**	*Asphodelustenuifolius* Cav.	Th	ANN
**Boraginaceae**	*Anchusaundulata* L.	Hem	PER
	*Asperugoprocumbens* L.	Th	ANN
	*Boragoofficinalis* L.	Th	ANN
	*Echiumhorridum* Batt.	Th	PER
	*Echiumhumile* Desf.	Hem	PER
	*Echiumplantagineum* L.	Th	ANN
	*Heliotropiumcrispum* Desf.	Ch	PER
	*Heliotropiumeuropaeum* L.	Th	ANN
	*Ogastemmapusillum* (Coss. & Durieu ex Bonnet & Barratte) Brummitt	Th	ANN
**Brassicaceae**	*Alyssum* sp.	Ch	PER
	*Anastaticahierochuntica* L.	Th	ANN
	*Biscutelladidyma* L.	Th	ANN
	*Diplotaxiscatholica* (L.) DC.	Th	ANN
	*Diplotaxistenuisiliqua* Delile	Th	ANN
	*Foleyolabillotii* Maire	Nph	PER
	*Hirschfeldiaincana* (L.) Lagr.-Foss.	Th	ANN
	*Matthiolaparviflora* W.T.Aiton	Th	ANN
	*Rapistrumrugosum* (L.) All.	Th	ANN
	*Zillaspinosa* (L.) Prantl	Nph	PER
**Capparaceae**	*Capparisspinosa* L.	Ch	PER
	*Cleomeamblyocarpa* Barratte & Murb.	Th	ANN
**Caprifoliaceae**	*Scabiosasemipapposa* Salzm. ex DC.	Th	ANN
**Caryophyllaceae**	*Herniariahirsuta* L.	Th	ANN
	*Paronychiaargentea* Lam.	Hem	PER
	*Polycarpontetraphyllum* (L.) L.	Th	ANN
	*Silenevulgaris* (Moench) Garcke	GR	ANN
	*Spergulapentandra* L.	Th	ANN
	*Spergulariabocconei* (Scheele) Graebner	Th	ANN
	*Stellariamedia* (L.) Vill.	Th	ANN
**Cistaceae**	*Helianthemumapenninum* (L.) Mill	Ch	PER
	*Helianthemumgetulum* Pomel	Ch	PER
**Compositae**	*Achilleasantolinoides* Lag.	Ch	PER
	*Aetheorhiza bulbosa subsp. bulbosa*	G	ANN
	*Anacycluspyrethrum* (L.) Lag.	Hem	PER
	*Anacyclusradiatus* Loisel.	Th	ANN
	*Anacyclusvalentinus* L.	Th	ANN
	*Asteriscusgraveolens* (Forssk.) Less.	Ch	PER
	*Atractyliscancellata* L.	Th	ANN
	*Brocchiacinerea* (Delile) Vis.	Th	ANN
	*Calendulaarvensis* (Vaill.) L.	Th	ANN
	*Calendula stellata Cav*.	Th	ANN
	*Carlinabrachylepis* (Batt.) Meusel & Kästner	Hem	PER
	*Centaureanapifolia* L.	Th	ANN
	*Centaureapullata* L.	Hem	PER
	*Centaureaseridis* L.	Hem	PER
	*Centaureasulphurea* Willd.	Th	ANN
	*Centranthusruber* (L.) DC.	Ch	PER
	*Cirsiumducellieri* Maire	Hem	PER
	*Cotulacoronopifolia* L.	Th	ANN
	*Crepisvesicaria* L.	Hem	PER
	*Cynaracardunculus* L.	G	PER
	*Cynarahumilis* L.	G	PER
	*Dittrichiaviscosa* (L.) Greuter	Ch	PER
	*Echinopsspinosissimus* Turra	Hem	PER
	*Filagohurdwarica* (Wall. ex DC.) Wagenitz	Th	ANN
	*Glebioniscoronaria* (L.) Cass. ex Spach	Th	ANN
	*Glebionissegetum* (L.) Fourr.	Th	ANN
	*Lactucaserriola* L.	Th	ANN
	*Lactucaviminea* (L.) J. Presl & C. Presl	Hem	PER
	*Launaeaarborescens* (Batt.) Murb.	Nph	PER
	*Mantisalcasalmantica* (L.) Briq. et Cavill.	Hem	PER
	*Pallenishierochuntica* (Michon) Greuter	Th	ANN
	*Pallenisspinosa* (L.) Cass.	Th	ANN
	*Phagnalonsaxatile* (L.) Cass.	Ch	PER
	*Pulicariaundulata* (L.) Kostel.	Ch	PER
	*Reichardiagaditana* (Willk.) Samp.	Th	ANN
	*Reichardiatingitana* Roth	Th	ANN
	*Schinusmolle* L.	Pha	PER
	*Scolymushispanicus* L.	Hem	PER
	*Seriphidiumherba-alba* (Asso) Y.R.Ling	Ch	PER
	*Silybummarianum* (L.) Gaertn.	Th	ANN
	*Sonchusasper* (L.) Hill	Th	ANN
	*Tolpisbarbata* (L.) Gaertn.	Th	ANN
	*Tolpisnemoralis* Font Quer	Hem	PER
	*Urospermumdalechampii* (L.) F. W. Schmidt	Hem	PER
	*Warioniasaharae* Benthem ex Benth. & Coss.	Nph	PER
**Convolvulaceae**	*Convolvulusalthaeoides* L.	Hem	PER
	*Convolvulusarvensis* L.	G	PER
**Crassulaceae**	*Umbilicus rupestris* (Salisb.) Dandy	G	PER
**Cucurbitaceae**	*Citrulluscolocynthis* (L.) Schrader	G	PER
**Euphorbiaceae**	*Euphorbianicaeensis* All.	Nph	PER
	*Euphorbiaofficinarum* L.	Nph	PER
**Fabaceae**	*Acaciasaligna* (Labill.) Wendl.	Ph	PER
	*Astragaluscaprinus* L.	Hem	PER
	*Ceratoniasiliqua* L.	Ph	PER
	*Crotalariasaharae* Cosson	Ch	PER
	*Ebenuspinnata* Aiton	Ch	PER
	*Hippocrepismultisiliquosa* L.	Th	ANN
	*Hypericumpubescens* Boiss.	Hem	PER
	*Lotusarenarius* Brot.	Th	ANN
	*Lotuscorniculatus* L.	Hem	PER
	*Lotuscreticus* L.	Ch	PER
	*Lotusmaroccanus* Ball	Hem	PER
	*Lupinusangustifolius* L.	Th	ANN
	*Medicagoorbicularis* (L.) Bartal.	Th	ANN
	*Medicagopolymorpha* L.	Th	ANN
	*Medicagorotata* Boiss.	Th	ANN
	*Melilotussulcatus* Desf.	Th	ANN
	*Ononisnatrix* L.	Ch	PER
	*Parkinsoniaaculeata* L.	Ph	PER
	*Retamamonosperma* (L.) Boiss.	Nph	PER
	*Tripodiontetraphyllum* (L.) Fourr.	Th	ANN
	*Vachelliatortilis* (Forssk.) Galasso & Banfi	Ph	PER
	*Viciasativa* L.	Th	ANN
**Frankeniaceae**	*Frankeniapulverulenta* L.	Th	ANN
**Gentianaceae**	*Centauriummaritimum* (L.) Fritsch	Th	ANN
**Geraniaceae**	*Erodiumbrachycarpum* (Godron) Thell.	Th	ANN
	*Erodiumcicutarium* (L.) L'Hér. ex Aiton,	Hem	ANN
	*Erodiummalacoides* (L.) L'Hér.	Th	ANN
	*Erodiumoxyrhinchum* M.Bieb.	Th	ANN
	*Geraniumrotundifolium* L.	Th	ANN
**Gisekiaceae**	*Gisekiapharnaceoides* L.	Th	ANN
**Juncaceae**	*Juncusacutus* L.	Hem	PER
**Lamiaceae**	*Ballotahirsuta* (Willd.) Benth.	Ch	PER
	*Lamiumamplexicaule* L.	Th	ANN
	*Lavandulamultifida* Burm.f.	Ch	PER
	*Marrubiumalysson* L.	Ch	PER
	*Marrubiumvulgare* L.	Ch	PER
	*Salviaaegyptiaca* L.	Ch	PER
	*Teucriumpolium* L.	Ch	PER
	*Teucriumspinosum* L.	Th	ANN
**Linaceae**	*Linumstrictum* L.	Th	ANN
	*Malvaparviflora* L.	Th	ANN
**Myrtaceae**	*Eucalyptus* sp.	Ph	PER
**Nitrariaceae**	*Nitrariaschoberi* L.	Ch	PER
	*Peganumharmala* L.	Ch	PER
**Orobanchaceae**	*Parentucellialatifolia* (L.) Caruel	Th	ANN
**Papaveraceae**	*Fumariaagraria* Lag.	Th	ANN
	*Glauciumflavum* Crantz	Hem	PER
	*Papaverrhoeas* L.	Th	ANN
**Plantaginaceae**	*Acanthorrhinumramosissimum* (Cosson & Durieu) Rothm.	Ch	PER
	*Misopatescalycinum* (Lange) Rothm.	Th	ANN
	*Plantagocoronopus* L.	Th	ANN
**Plumbaginaceae**	*Limoniumlobatum* (L.f.) Kuntze	Th	ANN
	*Plumbagoeuropaea* L.	Ch	PER
	*Saharanthusifniensis* (Caball.) M.B.Crespo & Lledó	Ch	PER
**Poaceae**	*Avenafatua* L.	Th	ANN
	*Bromussterilis* L.	Th	ANN
	*Cynodondactylon* (L.) Pers.	Hem	PER
	*Festuca* sp.	Th	ANN
	*Hordeummurinum* L.	Th	ANN
	*Hyparrheniahirta* (L.) Stapf	Hem	PER
	*Lamarckiaaurea* (L.) Moench	Th	ANN
	*Loliumrigidum* Gaudin	Th	ANN
	*Lygeumspartum* Loefl. ex L.	G	PER
	*Stipacapensis* Thunb.	Th	ANN
**Polygonaceae**	*Emexspinosa* (L.) Campd.	Th	ANN
	*Polygonumaviculare* L.	Th	ANN
	*Rumexvesicarius* L.	Th	ANN
**Portulacaceae**	*Portulacaoleracea* L.	Th	ANN
**Primulaceae**	*Lysimachiamonelli* (L.) U.Manns & Anderb.	Ch	PER
**Ranunculaceae**	*Adonismicrocarpa* DC.	Th	ANN
	*Delphiniumcossonianum* Batt.	Th	ANN
	*Delphiniumpentagynum* Lam	Th	ANN
	*Delphiniumperegrinum* L.	Th	ANN
**Resedaceae**	*Resedaalba* L.	Th	ANN
	*Resedalutea* L.	Th	ANN
**Rhamnaceae**	*Ziziphuslotus* (L.) Lam.	Nph	PER
**Rubiaceae**	*Cruciataarticulata* (L.) Ehrend.	Th	ANN
**Rutaceae**	*Rutamontana* (L.) L.	Th	ANN
**Scrophulariaceae**	*Scrophulariacanina* L.	Ch	PER
	*Verbascumpseudocreticum* Benedí & J.M.Monts.	Hem	PER
**Solanaceae**	*Daturastramonium* L.	Th	ANN
	*Hyoscyamusalbus* L.	Th	ANN
	*Hyoscyamusmuticus* L.	Th	ANN
	*Lyciumbarbarum* L.	Nph	PER
	*Nicotianaglauca* R.C. Graham	Nph	PER
	*Solanumdulcamara* L.	Hem	PER
	*Solanumherculeum* Bohs	Th	ANN
	*Solanumnigrum* L.	Th	ANN
	*Solanumsisymbriifolium* Lam.	Nph	PER
	*Withaniafrutescens* (L.) Pauquy	Nph	PER
**Tamaricaceae**	*Tamarixgallica* L.	Ph	PER
	*Tamarixaphylla* (L.) H.Karst.	Ph	PER
**Thymelaeaceae**	*Daphnegnidium* L.	Ch	PER
**Urticaceae**	*Forsskaoleatenacissima* L.	Th	ANN
	*Urticadioica* L.	GR	PER
**Zygophyllaceae**	*Zygophyllumgaetulum* Emb. & Maire	Ch	PER
	*Zygophyllumzilloides* (Humbert) Christenh. & Byng	Ch	PER
